# Effects of understory characteristics on browsing patterns of roe deer in central European mountain forests

**DOI:** 10.1002/ece3.10431

**Published:** 2023-08-14

**Authors:** Sebastian Schwegmann, Martin Mörsdorf, Manisha Bhardwaj, Ilse Storch

**Affiliations:** ^1^ Chair of Wildlife Ecology and Management University of Freiburg Freiburg Germany; ^2^ Chair of Geobotany University of Freiburg Freiburg Germany

**Keywords:** associational effects, browsing, *Capreolus capreolus*, deadwood, silver fir, understory

## Abstract

Selective browsing by deer on young trees may impede the management goal of increasing forest resilience against climate change and other disturbances. Deer population density is often considered the main driver of browsing impacts on young trees, however, a range of other variables such as food availability also affect this relationship. In this study, we use browsing survey data from 135 research plots to explore patterns of roe deer (*Capreolus capreolus*) browsing pressure on woody plants in mountainous forests in central Europe. We fitted species‐specific generalised linear mixed models for eight woody taxa, assessing the potential effects of understory characteristics, roe deer abundance and lying deadwood on browsing intensity. Our study reveals conspecific and associational effects for woody taxa that are intermediately browsed by roe deer. Selective browsing pressure was mediated by preferences of plants, in that, browsing of strongly preferred woody taxa as for example mountain ash (*Sorbus aucuparia*) and of least preferred woody taxa, for example Norway spruce (*Picea abies*) was not affected by the surrounding understory vegetation, while browsing pressure on intermediately browsed species like for example silver fir (*Abies alba*) was affected by understory characteristics. Contrary to our expectations, roe deer abundance was only positively associated with browsing pressure on silver fir and bilberry (*Vaccinium myrtillus*), while all other plants were unaffected by deer abundance. Finally, we did not find an influence of lying deadwood volume on the browsing pressure on any woody‐plant species. Overall, our results indicate that patterns in browsing preference and intensity are species‐specific processes and are partly affected by the surrounding understory vegetation. Current management strategies that aim to reduce browsing pressure through culling may be inefficient as they do not address other drivers of browsing pressure. However, managers also need to consider the characteristics of the local understory vegetation in addition to deer abundance and design species‐specific plans to reduce browsing on woody plant taxa.

## Graphical abstract

We assessed the associational effects of characteristics of the herb layer cover on the browsing pressure of roe deer on woody plants. We found associational effects for woody taxa that were of intermediate preference to deer, while roe deer abundance only affected browsing pressure on few woody plants. Overall, associational effects of understory characteristics appear to be highly species‐specific and context‐dependent.

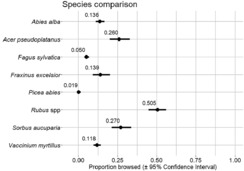

## 1 INTRODUCTION

Browsing on young trees by ungulates can be problematic for forest managers who are motivated to increase the resilience of forests towards climate change. In central Europe, large amounts of Norway spruce (*Picea abies* (L.) H.Karst.) stands have been destroyed by bark beetle infestations in the last decade (Biedermann et al., 2019). While bark beetle calamities are natural disturbances, the frequency of infestation events is increasing due to climate change (Bentz et al., 2010; Cudmore et al., 2010). One way to make forests more resilient to disturbances, such as climate change and frequent pest infestations, is to increase tree species richness (Berthelot et al., 2021; Jactel et al., 2017). In mountainous regions of central Europe, this is usually achieved by increasing the proportions of silver fir (*Abies alba* MILL.) and deciduous tree species in the canopy (Lebourgeois et al., 2013; Schwarz & Bauhus, 2019). However, efforts to improve heterogeneity in forests are undermined by growing deer abundances, which increase the extent to which young trees are browsed (Häsler & Senn, 2012; Moser et al., 2006; Senn & Suter, 2003). Deer browsing reduces the survival of young trees (Ammer, 1996), delays regeneration (Kupferschmid et al., 2019), and can reduce tree species diversity through selective browsing (Perea et al., 2014; Ward & Williams, 2020). Consequently, browsing by large herbivores might inhibit the development of forest resilience (e.g. against climate change) by affecting long‐term tree species composition (Champagne et al., 2021; Meier et al., 2017).

Roe deer (*Capreolus capreolus* L.) are the most common and widespread ungulates in central Europe (Lorenzini et al., 2022). As concentrate selectors (Barančeková et al., 2010; Tixier & Duncan, 1996), roe deer are highly adaptable in their feeding behaviour (Dahl et al., 2020; König et al., 2020), and consume a variety of vegetation types. The proportion of woody plant consumption in roe deer diet depends on the availability of other forage in their environment. For example, woody plants make up a greater proportion of roe deer diet in the winter compared to the summer because there are fewer non‐woody plants available in winter (Barančeková et al., 2010; Häsler & Senn, 2012; Tixier & Duncan, 1996). As roe deer populations become more abundant, the overall browsing pressure in forest systems increases (Borowski, Gil, et al., 2021; Hothorn & Müller, 2010; Tremblay et al., 2007). However, where and how intensely the browsing occurs is influenced by factors such as forest structure (Kupferschmid et al., 2020), predation pressure (Kuijper et al., 2013), anthropogenic disturbance (Borowski, Bartoń, et al., 2021; Gerhardt et al., 2013; Möst et al., 2015) and surrounding land‐use (Takarabe & Iijima, 2020).

The availability of forage influences the browsing intensity on individual plants through associational effects (Hagen & Suchant, 2020; Moser et al., 2006; Skoták et al., 2021; Underwood et al., 2014; Ward et al., 2008). In the case of ‘associational susceptibility’, the availability of other potential‐forage plants increases the overall attraction of a vegetation patch, thus intensifying the browsing pressure on individual plants (Milligan & Koricheva, 2013; Vehviläinen et al., 2007). Where individual young trees are browsed on less because the browsing pressure is deflected to other available forage, the composition of the understory reduces browsing damage on woody vegetation, demonstrating ‘associational resistance’ (Champagne, Moore, et al., 2018; Milligan & Koricheva, 2013). The probability of an individual woody plant being browsed is also affected by proximity and abundance of conspecifics (‘conspecific effects’). Thus, the abundance of forage may motivate habitat/patch selection, while the diversity of the understory might affect browsing pressure on individual plants (Häsler & Senn, 2012), and the likelihood of an individual plant being browsed is affected by the presence of conspecifics (Champagne et al., 2020; Otway et al., 2005; Underwood et al., 2014). Browsing intensity has been previously described in relation to the diversity of woody vegetation (Ameztegui & Coll, 2015; Ohse et al., 2017; Ward et al., 2008), however, the role of associational effects and conspecific effects on the intensity by which woody plants are browsed understory is understudied.

Browsing damage is typically mitigated through culling of herbivores because abundance and access of individuals to forage is thought to be the main driver of browsing intensity (Hothorn & Müller, 2010). In some cases, reducing herbivore density and abundance is a successful intervention (Jenkins et al., 2014), however, often, hunting alone does not reduce browsing damage sufficiently (Kamler et al., 2010; Wright et al., 2012). Counterproductively, high and continuous hunting pressure can even increase damage by deer locally because deer use local resources more intensively to avoid risks through movement (Gerhardt et al., 2013; Nopp‐Mayr et al., 2011). The relationship between herbivore abundance and browsing pressure is not yet fully understood and requires further investigation.

Retention forestry has emerged as a practice to conserve forest biodiversity within production forests, by retaining old‐growth features like deadwood and old trees (Gustafsson et al., 2020). Next to contributing to the conservation of saproxylic species, the retention of lying deadwood may indirectly facilitate the natural succession of young trees through physical protection from herbivore access (Pellerin et al., 2010; Whyte & Lusk, 2019). While the effectiveness of retaining deadwood on biodiversity conservation has been explored, less is known about the effect of deadwood on mitigating browsing pressure on woody vegetation (Hagge et al., 2019; Hall Defrees et al., 2021; Pellerin et al., 2010; van Ginkel et al., 2021).

In this study, we aimed to assess the potential associational effects of understory vegetation on the browsing impact of roe deer in a central European mountainous forest system managed using retention forestry methods. In particular, we were interested in how characteristics of the understory vegetation, relative roe deer abundance and lying deadwood influenced browsing on woody plants by roe deer. We aimed to answer the following questions:

1. What are the associational effects of understory diversity, cover, or the abundance of woody plants on the browsing pressure of roe deer on woody plants?
2. What are the conspecific effects, for example abundance of or proximity to conspecifics, driving browsing pressure of wood plants?
3. What influence does relative roe deer abundance have on browsing pressure?
4. What influence does lying deadwood have on browsing pressure?

Insights from this study can strengthen our knowledge of the context‐dependency of foraging pressure, helping forest managers understand how best to manage ground vegetation in regenerating stands when taking measures against browsing damage. To our knowledge this is the first study assessing the above‐mentioned questions for central European forests, only exposed to roe deer browsing, while previous studies analyse the effect of multiple deer species. Knowledge of browsing patterns in central Europe is especially relevant as forest management adopts *close‐to‐nature* strategies like retention forestry that incorporate measures of biodiversity conservation into forestry.

## 2 METHODS

### 2.1 Study area

We conducted this study in the southern Black Forest in south‐western Germany (Latitude: 47.6°–48.3° N, Longitude: 7.7°–8.6° E, WGS 84); a low mountain range covered with forests that are interspersed with villages and open land. Since 2016, 135 one‐hectare research plots within the southern Black Forest have been systematically surveyed to explore the relationship between forest biodiversity, for example richness and abundance of available fauna and flora, and retention forestry (*ConFoBi – Conservation of Forest Biodiversity in Multiple‐use Landscapes of Central Europe*; Storch et al., 2020). All plots (443–1334 m a.s.l.) are covered by forest stands dominated by Norway spruce, silver fir and European beech (*Fagus sylvatica* L.) and were chosen based on gradients of standing deadwood and forest fragmentation in mayor forest stands (>60 years), with at least 760 m distance between each other.

Roe deer are the most abundant large herbivores in the region, while chamois (*Rupicapra rupicapra* L.) occur in the high elevations of the Black Forest. Red deer (*Cervus elaphus* L.) are localised to designated red deer areas, which were excluded from the selected plots, while introduced Sika deer (*Cervus nippon* TEMMINCK) occur in low numbers. All large herbivores are hunted to control population size.

### 2.2 Browsing survey

**FIGURE 1:**
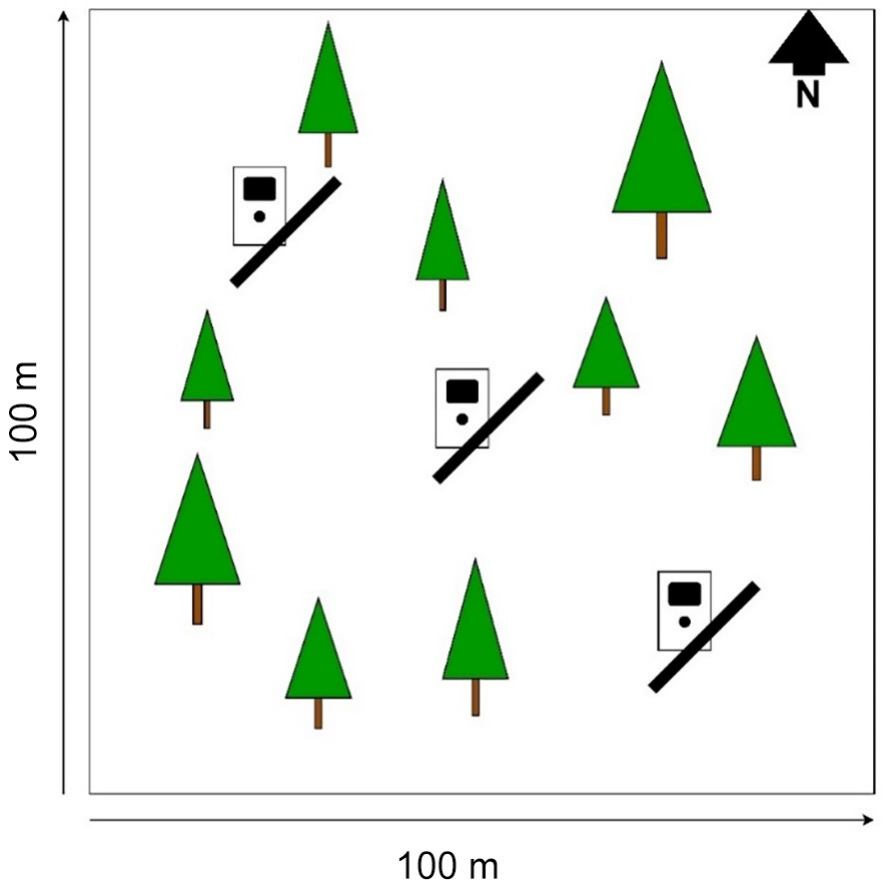
Schematic of methodological set‐up of 2 × 15 m^2^ browsing transects (black bars) and camera trap positions on one‐hectare research plots.

We conducted browsing surveys on all 135 research plots in autumn (October–November) 2019 (*n* = 71) or 2020 (*n* = 64) to assess summer browsing on woody plants. In the following spring (March–April), we repeated the surveys to assess browsing intensity over the full year. We assessed all woody vegetation between 8 and 130 cm of height, including young trees, shrubs, and dwarf‐shrubs, within 1 m of each side of three 15 m long transects (Figure 1). Assessment included recording the species, height, and position in relation to conspecifics (Grouping, Table 1). We counted the number of trees from each species on the transect, and for each tree species, we surveyed up to 20 individuals for signs of browsing. For shrubs in clonal colonies, each ramet was treated as an individual, as true separation of ramets was impossible to determine without removing the plant from the soil. As such, for *Rubus* spp. and *Vaccinium myrtillus* (L.), percent of coverage was estimated instead of counting individuals. If more than 20 individuals were found in a transect, we aimed to sample a set of individuals spread over the entire length of the transect as well as along the entire range of height and grouping constellations. We assigned each individual an index of browsing intensity, which was the proportion of browsed branches of the 10 highest branches. If the plant had less than 10 branches, all branches were assessed. Browsing transects were positioned at fixed points in the northwest corner, the centre, and the southeast corner of the plots (Figure 1). Transects ran from the southwest to northeast, unless plots had a distinct slope, in which case transects were always parallel to the slope. To account for overall forage availability, we estimated the percentage of cover of understory vegetation (UnderC), including young trees, dwarf shrubs, herbs, grasses, sedges, and ferns within 5 m of the transects. Other vegetation‐related variables assessed were: the total number of young trees in the transect (TotalWP), the total number of conspecifics in the transect and the plot‐level diversity of the understory (UnderDiv, Table 1).

**TABLE 1:** Table of predictor variables used in the species‐specific models.

Variable	Description	Unit	Scale	Mean (SD)
RDeer	Average number of roe deer detections per trapnight over five camera trapping periods from Spring 2019 to Spring 2021.	Events per trap‐night	Plot	0.236 (0.172)
UnderC	Cover of understory vegetation including herbs, grass, sedges, fern as well as young trees and shrubs (<130 cm).	%	Within 5 m of transect	31.753 (21.943)
TotalWP	Total count of young trees in the transect. Individuals of dwarf shrubs were not included.	Count	Within 1 m of Transect	50.390 (58.932)
UnderDiv	Shannon index of understory diversity on the plot (from Helbach et al., 2022). Aggregated from multiple subplots.	Index	Plot	2.443 (0.586)
Grouping	Position of the individual plant in relation to conspecifics.	Category	Individual plant	Single: 6065 individuals
	Single: standing alone			Grouped: 4511 individuals
	Group: individual is part of a group with at least one other conspecific with overlapping or touching leaves or branches.			
Conspecifics	Number of conspecifics counted in the transect for tree species. For shrubs cover (%) in the wider transect was used.	Count	Individual plant	Trees: 32.861 (49.992)
				Shrubs: 15.560 (16.526)
Deadwood	Volume of lying deadwood on the research plot, assessed in a full inventory in 2018 (Storch et al., 2020).	m^3^/ha	Plot	36.241 (35.550)
Height	Height of the individual plant.	cm	Individual plant	33.954 (23.806)
Year	Survey year 2019/2020 or 2020/2021.	Category	Not applicable	19/20: 5890 individuals
				20/21: 4686 individuals

### 2.3 Camera trapping

To assess the relative abundance of roe deer (RDeer), we used detection rates of camera traps, collected in five camera trapping periods, during spring in 2019–2021, and autumn in 2019–2020 (Rovero & Marshall, 2009). In every camera trapping period, we placed one camera trap close to one of the three transects (Figure 1). We assigned the first position randomly and henceforth systematically shifted within these three positions through the five seasons. For a detailed description of the camera trap setup see Schwegmann et al. (2023). We summed counts of roe deer events per season and corrected for trapping effort. Roe deer events were deemed independent when detections were at least 5 minutes apart. For this study, the mean number of events per trapnight, averaged over all five survey periods, was used because we assumed that roe deer use of the plots would not significantly change between seasons (Appendix S1).

### 2.4 Statistical analysis

For each woody‐plant species, we assessed the impact of surrounding understory characteristics on browsing intensity. We calculated species‐specific models for woody‐plant species with data from more than 200 individuals: *Abies alba*, *Picea abies*, *Fagus sylvatica*, *Sorbus aucuparia* (L.), *Acer pseudoplatanus* (L.), *Fraxinus excelsior* (L.), *Vaccinium myrtillus* and *Rubus* spp. To assess the factors influencing browsing pressure and the difference in browsing intensity between species over the whole year, we used the data from the spring survey.

For each woody‐plant species, we fitted separate generalised linear mixed models (glmm) for each individual plant, with browsing intensity index as the response. We assumed a binomial data distribution as we used proportional data derived from counts (Douma & Weedon, 2019). All continuous variables were scaled. In every model, we used the same set of explanatory variables (Table 1). We used the height of each individual plant with a quadratic effect, to account for a potential optimal browsing height. To assess the potential effect of deadwood on roe deer browsing intensity by obstructing deer movement we added the variable *Deadwood* describing the volume of lying deadwood on the scale of the research plots (Table 1). Finally, we included a nested random effect for transect within research plot to account for spatial dependence of woody plants within the transect and the plot.

Following data inspection according to Zuur et al. (2010), we excluded total woody plants from the models for beech and spruce, due to collinearity with a number of conspecifics (Dormann et al., 2013). The variable Grouping was dropped from the species‐specific model for *F. excelsior* as no individual in close groups were surveyed, thereby resulting in only one category. We selected all best‐fitting candidate models with an ∆AICc < 2 and report the resulting conditional averaged models. Results were deemed to be significant when alpha was 0.05 or smaller. Model assumptions were visually assessed.

To compare the browsing intensity among species without the possible effects of other variables, we fitted another glmm, using species as a variable and influential predictors from the species‐specific models as random effects (Height, UnderC, UnderDiv, and Year). To assess the difference in browsing intensity between summer and the full year we used the Wilcoxon ranked‐sum test to compare the browsing intensity from the total autumn and spring survey. Surveys of autumn and spring were not truly independent, and the unequal sample size did not allow us to use a paired test. We performed all analyses in R 4.1.2 (R Core Team, 2021). For the species‐specific glmms, we used the *glmmTMB* function (Brooks et al., 2017), while model selection was conducted using *MuMIn* (Barton, 2020).

## 3 RESULTS

**FIGURE 2:**
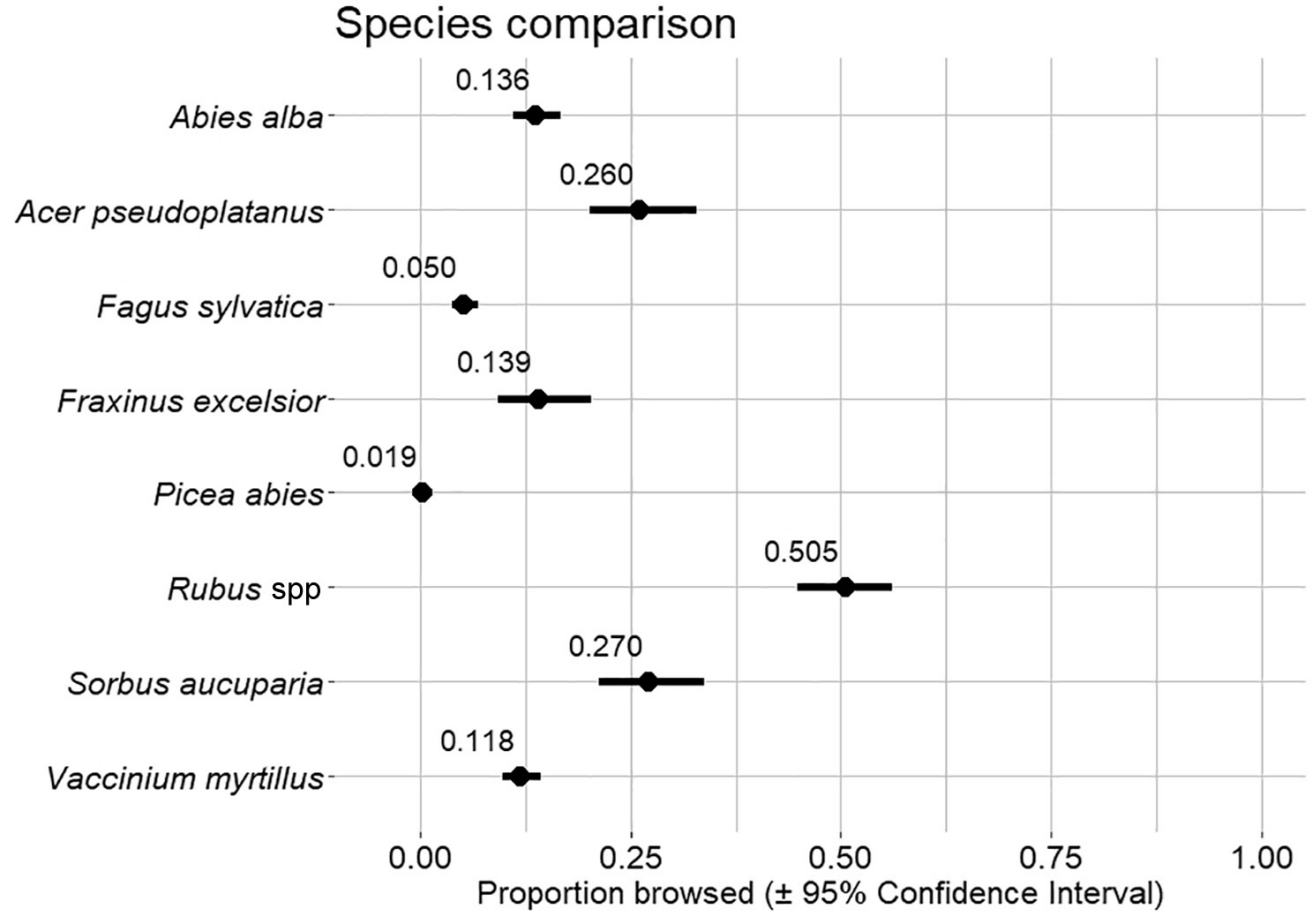
Comparison of browsing intensity among woody‐plant species, derived from glmm comparing browsing intensity of all woody plant taxa. Based on transformed values from glmm correcting for plant height and vegetation features of the understory.

We sampled a total of 11,123 and 10,576 individual woody plants from autumn and spring surveys respectively, belonging to seven species and one additional genus (Table 2). *P. abies* was the most abundant woody plant, with 2959 individuals surveyed during spring surveys and *F. excelsior* had the least individuals (251 in spring). *Rubus* spp. was, with 50.5% of branches browsed in spring (corrected through glmm comparing all species; absolute value 45.8%; Table 2; Figure 2), the most intensively browsed taxa, while *P. abies* with 1.9% was least browsed (0.3% uncorrected). Among tree species, browsing pressure in spring was highest for *A. pseudoplatanus* with 26% of branches browsed (29.6% uncorrected) and *S. aucuparia* with 27% (27.6% uncorrected), followed by *F. excelsior* with 13.9% (17.3% uncorrected) and *A. alba* with 13.6% (16.6% uncorrected) branches browsed. For all taxa but *P. abies*, there was a significant increase in browsing between the autumn and spring surveys (Table 2). The increase in browsing between autumn and spring ranged from 33.33% for *P. abies* to 62.66% for *Rubus* spp. The average increase in browsing between autumn and spring for all 8 taxa included in the analysis was 45.49%.

**TABLE 2:** Results of browsing survey by woody‐plant species.

Species	Autumn			Spring			Percent of browsing in winter	Wilcoxon test
	*N*	Mean (SD)	Proportion branches browsed	*N*	Mean (SD)	Proportion branches browsed		
*Abies alba*	1473	0.083 (0.178)	0.263	1474	0.166 (0.290)	0.355	50.00	*W* = 1,212,868, *p* < .001
*Acer pseudoplatanus*	356	0.181 (0.284)	0.368	374	0.296 (0.340)	0.540	38.85	*W* = 79,458, *p* < .001
*Fagus sylvatica*	996	0.038 (0.114)	0.157	956	0.072 (0.157)	0.288	47.22	*W* = 537,998, *p* < .001
*Fraxinus excelsior*	239	0.089 (0.222)	0.176	251	0.173 (0.308)	0.311	48.55	*W* = 34,195, *p* < .001
*Picea abies*	2815	0.002 (0.025)	0.008	2959	0.003 (0.036)	0.010	33.33	*W* = 4,174,563, *p* = .346
*Rubus* spp.	2086	0.171 (0.359)	0.359	1398	0.458 (0.413)	0.634	62.66	*W* = 2,018,464, *p* < .001
*Sorbus aucuparia*	313	0.198 (0.293)	0.396	410	0.276 (0.372)	0.429	28.26	*W* = 752,331, *p* < .001
*Vaccinium myrtillus*	2845	0.063 (0.159)	0.189	2754	0.140 (0.260)	0.307	55.00	*W* = 385,914, *p* < .001

*Note*: We present the sample size per species (*N*), uncorrected mean and standard deviation of branches browsed (mean (SD)), and proportion of branches browsed on each individual plant (proportion branches browsed). Autumn survey reflects browsing from summer of the same year, while the spring survey assessed browsing in the previous summer and spring. Percent of browsing in winter is the change in browsing between subsequent surveys, stated as a percentage increase. Wilcoxon test gives the results comparing the browsing intensity between autumn and spring.

### 3.1 Drivers of browsing pressure

The results of the species‐specific models represent the conditional averaged model results assessing the effects of understory vegetation, roe deer abundance and deadwood on roe deer browsing pressure on woody plants (Table 3). All included candidate models can be found in Appendix S2.

**TABLE 3:** Conditionally averaged model results describing the factors which influences the likelihood for an individual tree to be browsed.

	*Abies alba*				*Acer pseudoplatanus*				*Fagus sylvatica*				*Fraxinus excelsior*			
	Estimate	Std‐error	*z*‐Value	*p*‐Value	Estimate	Std‐error	*z*‐Value	*p*‐Value	Estimate	Std‐error	*z*‐Value	*p*‐Value	Estimate	Std‐error	*z*‐value	*p*‐Value
Intercept	**−2.911**	**0.273**	**10.660**	**<.001**	**−1.049**	**0.257**	**4.072**	**<.001**	**−2.680**	**0.201**	**13.291**	**<.001**	**−1.523**	**0.277**	**5.485**	**<.001**
Rdeer	**0.263**	**0.104**	**2.531**	**.011**	**−0.454**	**0.142**	**3.178**	**.001**	0.120	0.111	1.082	.279	−0.202	0.195	1.029	.303
Height	**2.336**	**0.363**	**6.428**	**<.001**	0.676	0.526	1.283	.200	0.010	0.417	0.841	.981	0.996	0.776	1.281	.200
Height^2^	**−1.982**	**0.384**	**5.152**	**<.001**	−0.608	0.525	1.155	.248	−2.664	0.317	0.024	.400	−0.900	0.798	1.124	.261
TotalWP	**0.421**	**0.141**	**2.973**	**.003**	−0.052	0.117	0.448	.654					−0.220	0.250	0.875	.381
UnderC	**−0.352**	**0.128**	**2.742**	**.006**	0.028	0.119	0.235	.814	0.107	0.128	0.835	.404	**−0.781**	**0.297**	**2.623**	**.009**
UnderDiv	**0.406**	**0.128**	**3.162**	**.002**	−0.068	0.120	0.563	.573	−0.092	0.133	0.692	.489	−0.490	0.303	1.611	.107
Grouping Single	**0.561**	**0.242**	**2.313**	**.021**	0.333	0.657	0.505	.613	0.135	0.303	0.446	.656				
Conspecifics	**−0.330**	**0.168**	**1.967**	**.049**					−0.472	0.267	1.767	.077	0.410	0.241	1.697	.090
Deadwood	0.102	0.111	0.924	.355	−0.114	0.125	0.909	.364	0.045	0.119	0.379	.704	−0.258	0.258	0.996	.319
Year 2020/2021	**0.608**	**0.242**	**2.510**	**.012**	0.213	0.242	0.876	.381	0.091	0.256	0.353	.724	−0.814	0.661	1.228	.219

	*Picea abies*				*Rubus* spp.				*Sorbus aucuparia*				*Vaccinium myrtillus*			
	Estimate	Std‐error	*z*‐Value	*p*‐Value	Estimate	Std‐error	*z*‐Value	*p*‐Value	Estimate	Std‐error	*z*‐Value	*p*‐Value	Estimate	Std‐error	*z*‐Value	*p*‐Value
Intercept	**−7.845**	**1.147**	**6.837**	**<.001**	−0.103	0.201	0.510	.610	**−1.679**	**0.227**	**7.361**	**<.001**	**−2.264**	**0.146**	**15.438**	**<.001**
Rdeer	0.286	0.348	0.821	.411	0.121	0.126	0.940	.347	0.137	0.149	0.920	.357	**0.396**	**0.093**	**4.241**	**<.001**
Height	0.967	1.180	0.819	.413	**2.547**	**0.236**	**10.774**	**<.001**	**1.941**	**0.572**	**3.385**	**<.001**	**0.928**	**0.291**	**3.192**	**.001**
Height^2^	−0.594	1.280	0.464	.643	**−1.589**	**0.221**	**7.168**	**<.001**	**−1.676**	**0.566**	**2.960**	**.003**	**−0.688**	**0.291**	**2.363**	**.018**
TotalWP					−0.181	0.132	1.371	.170	−0.195	0.162	1.201	.230	**−0.357**	**0.127**	**2.798**	**.005**
UnderC	0.390	0.399	0.976	.329	−0.203	0.139	1.458	.145					**0.392**	**0.138**	**2.835**	**.005**
UnderDiv	0.197	0.475	0.413	.679	0.035	0.124	0.284	.776	0.093	0.140	0.661	.508	0.119	0.117	1.020	.308
Grouping Single	−0.538	0.733	0.734	.463	−0.219	0.183	1.200	.230					−0.188	0.161	1.165	.244
Conspecifics	−1.662	1.322	1.257	.209	−0.244	0.164	1.489	.137	−0.246	0.163	1.509	.131	**−0.540**	**0.157**	**3.449**	**<.001**
Deadwood	0.220	0.244	0.902	.367	0.079	0.126	0.632	.528	−0.219	0.163	1.342	.179	−0.096	0.101	0.952	.341
Year 2020/2021	−0.731	0.978	0.747	.455	−0.017	0.262	0.064	.949	**1.283**	**0.321**	**3.984**	**<.001**	0.150	0.199	0.752	.452

*Note*: Missing variables are those that were not identified within the top‐performing models (∆ AICc ≤2) selected for each individual species model. Variables with a significant effect (α = 0.05) are bold.

Most species–variable relationships in our tests were not significant, however, there are a few notable exceptions. Increasing understory cover resulted in decreased browsing of *A. alba* and *F. excelsior* (*p* = .006 and *p* = .009, respectively), but increased browsing of *V. myrtillus* (*p* = .005). Increasing the total number of woody plants resulted in decreased browsing on *V. myrtillus* (*p* = .005), but increased browsing on *A. alba* (*p* = .003). As understory diversity increased, so did browsing on *A. alba* (*p* = .002), however, no other species was responsive towards understory diversity. Increased abundance and proximity of conspecifics affected browsing on *A. alba* and *V. myrtillus*, in that *A. alba* was browsed more when growing alone (*p* = .021), while the abundance of conspecifics reduced browsing pressure on *V. myrtillus* and *A. alba* (*p* < .001 and *p* = .049). Roe deer abundance was positively related to browsing intensity on *A. alba* (*p* = .011) and *V. myrtillus* (*p* < .001), but negatively affected browsing intensity on *A. pseudoplatanus* (*p* = .001). The volume of lying deadwood was not related to browsing intensity for any of the investigated woody‐plant species. Linear and quadratic terms of individual plant height affected roe deer browsing intensity on *A. alba*, *Rubus* spp., *S. aucuparia* and *V. myrtillus* significantly in a humped‐shaped curve (*p* < .05), with the lowest browsing intensity at high and low plant heights.

## 4 DISCUSSION

The influence of understory characteristics through associational and conspecific effects on browsing intensity by roe deer varied between woody plant species. We found associational and conspecific effects for species that were browsed with intermediate intensity by roe deer (Question 1 and 2). *F. excelsior* was browsed less as overall understory cover increased, while browsing of *V. myrtillus* increased with understory cover, but decreased with the abundance of wood plants and number of conspecifics. Browsing of *A. Alba* decreased with understory cover and proximity to conspecifics and increased with understory cover and diversity. Browsing of *V. myrtillus*, and *A. Alba* increased with relative roe deer abundance, but roe deer abundance had a negative impact on browsing of *A pseudoplatanus* (Question 3). Finally, lying deadwood had no effect on the browsing pressure of any plant species (Question 4).

While we did not assess the preference of each plant species by roe deer directly, the proportions of branches browsed per species align with what has been found in other studies about roe deer preferences: *Rubus* spp., followed by *A. pseudoplatanus* and *S. aucuparia* are highly preferred (i.e. heavily browsed in proportion to available Kupferschmid et al., 2020; Moser et al., 2006; Szwagrzyk et al., 2020), while *P. abies* and *F. sylvatica* are avoided by roe deer (i.e. not often browsed, despite availability Boulanger et al., 2009; Mattila & Kjellander, 2016). *A. alba*, *F. excelsior* and *V. myrtillus* were intermediately browsed in relation to the other species (Häsler & Senn, 2012; Kupferschmid et al., 2020; Senn & Suter 2003; Szmidt, 1975).

### 4.1 Associational effects through diversity and cover

Overall, we found associational effects of the understory for *A. alba*, *F. excelsior* and *V. myrtillus*, but not on woody plants of high (*Rubus* spp., *S. aucuparia*, *A. pseudoplatanus*) and very low (*P. abies*, *F. sylvatica*) preference for roe deer. Individuals of these species are either always browsed upon, or always avoided by roe deer independent of the characteristics of the understory.

High Shannon‐diversity of the understory increased the proportion of *A. alba* branches browsed indicating associational susceptibility but had no effect on other woody plant taxa. We assume that increased plant diversity also increases the availability and diversity of attractive forage plants for roe deer (Barančeková et al., 2010; Ohse et al., 2017). Roe deer, as concentrate selectors, prefer to browse on a variety of food plants, and a higher patch diversity may increase the attractivity of the site which leads to a high site use by roe deer. Consequently, proximity to attractive resources increases the opportunistic use of a less attractive resource and thus leads to associational susceptibility (Bergvall et al., 2006; Courant & Fortin, 2010; Häsler & Senn, 2012). Other studies found contrasting effects of plant diversity on browsing intensity of individual focal plants. Similar to our study, Vehviläinen and Koricheva (2006) and Milligan and Koricheva (2013) found an increase of browsing pressure with higher patch diversity, while Champagne, Dumont, et al. (2018) and Ohse et al. (2017) found reduced browsing on sites with high Shannon diversity or species richness respectively. Overall, the effect of understory diversity depends likely on the species identity of the occurring plants and their respective quality as forage plants, but probably also their availability on larger spatial scales. In general, the abundance of high‐quality forage plants will generally increase attraction of herbivores to the site and thus increase herbivory (Bee et al., 2009; Champagne et al., 2020; Ohse et al., 2017; Wang et al., 2010). Due to differences in feeding preferences, food‐niche width and selectivity, the associational effects resulting from high or low forage quality are herbivore‐specific (Barbosa et al., 2009; Champagne, Dumont, et al., 2018; Vehviläinen & Koricheva, 2006).

Woody plant abundance had contrasting effects on roe deer browsing intensity, namely more woody plants increased browsing pressure on *A. alba* and decreased browsing pressure on *V. myrtillus*, which might be due to multiple effects. High abundances of woody plants specifically may provide increased hiding cover as well as protection from environmental conditions in addition to forage and may increase site selection for roe deer (Bobrowski et al., 2020; Gill et al., 1996; Ohse et al., 2017), consequently leading to browsing susceptibility of other plants. However, woody plants can also physically protect neighbouring trees from browsing, thus functioning as nursing plants (Ameztegui & Coll, 2015; Gómez‐Aparicio et al., 2008; Smit et al., 2007). In this case, the contrasting directionalities might be due to species identity or physical structure of the woody vegetation, for example, reduced browsing on *V. myrtillus* could be due to higher preference of roe deer for other woody plants. For comparison, Champagne, Dumont, et al. (2018) found that a higher number of available shoots increased browsing pressure.

Although habitats with high understory cover are attractive for roe deer (Heinze et al., 2011; Tufto et al., 1996), the understory cover can deflect browsing from individual *A. alba* and *F. excelsior*. Higher forage availability may lead to reduced selection of specific plants for browsing, however, the current literature does not always support this idea. For example, Kupferschmid et al. (2020) found an increase in browsing on seedlings with higher understory cover, while other studies found a decrease in browsing with higher understory cover (Verheyden‐Tixier et al., 1998), or no effect (Bergquist & Örlander, 1998). The discrepancies are likely influenced by confounding effects, such as herbivore species studied, its feeding behaviour (i.e. browser or grazer) and the composition large herbivore community. Less selective herbivores than roe deer (e.g. red deer, Gebert & Verheyden‐Tixier, 2001) might for example, select vegetation patches due to the available volume of forage and less for its quality, leading associational susceptibility for individual woody plants. In our study, *V. myrtillus* was browsed more when understory cover increased. *V. myrtillus* is not of high preference for roe deer, but is still an important food source due to its general high availability and its tendency to outcompete other plants in the understory (Barančeková et al., 2010; Petersson et al., 2019; Tixier & Duncan, 1996). Thus, sites with high overall understory cover, with other more attractive resources may lead to associational susceptibility for *V. myrtillus*.

### 4.2 Conspecific effects

Overall, we found conspecific effects for *A. alba* and *V. myrtillus*. For *A. alba* grouping with conspecifics reduced browsing impact, showing conspecific resistance, likely due to a dilution effect (Champagne et al., 2020). Furthermore, the abundance of conspecifics reduced browsing intensity on *A. alba* and *V. myrtillus*. This may further support that *A. alba* and *V. myrtillus* are not a highly sought‐after forage plants for roe deer, because if it was, grouping of very attractive forage plants should increase browsing pressure on the site (Bee et al., 2009; Vehviläinen & Koricheva, 2006). We cannot see this effect for the species of high preference in this study, probably because such species (i.e. *S. aucuparia* and *A. pseudoplatanus*) infrequently grow in groups of conspecifics. Similarly, we cannot find an effect for *Rubus* spp., possibly because it is overall very abundant and therefore probably does not affect the selection of vegetation patches by roe deer.

### 4.3 Relevance of roe deer abundance

Abundance of herbivores is often thought to drive browsing pressure on plants (Borowski, Gil, et al., 2021; Klopcic et al., 2010; Ward & Williams, 2020). Contrary to our expectations, we found that this is only true for *A. alba* and *V. myrtillus*, while roe deer abundance did not increase the browsing of any other plant species (similar to Kamler et al., 2010; Wright et al., 2012). While culling of deer can help reduce browsing pressure (Hothorn & Müller, 2010; Ward & Williams, 2020), it may only help certain woody‐plant species, specifically those that are not of high interest to deer (Kamler et al., 2010). Additionally, our results show that using browsing indices as a proxy for ungulate density (e.g. in management) may only be an imprecise measure, weakly related to actual ungulate abundance.

### 4.4 The impact of lying deadwood

We did not find evidence that lying deadwood affected roe deer browsing intensity on woody plants. Lying deadwood has been suggested to pose a physical barrier for deer and thus locally reduces browsing on woody plants (Hagge et al., 2019), however, the results in the literature are not consistent (e.g. Kupferschmid et al., 2020; Pellerin et al., 2010). In our own study system, we previously demonstrated localised site avoidance by roe deer in autumn where deadwood was abundant (Schwegmann et al., 2023), but in the present study, we cannot demonstrate reduced browsing intensity with increasing deadwood volume. The volumes of deadwood on our study sites (on average 36.24 m^3^/ha, total range 0–297 m^3^/ha) are significantly lower than reported from natural or primaeval montane beech‐fir forests (on average 223.9 m^3^/ha) (Bujoczek et al., 2018). The relatively low amounts of deadwood on our research sites might mediate roe deer habitat use, but not physically inhibit browsing once deer are present. Thus, the potential lower browsing impact would already be explained by relative roe deer abundance in this study. Future studies should investigate the relationship between deadwood and browsing in more detail considering multiple spatial scales as well as a wider gradient in lying deadwood volume. Forest managers possibly need to retain larger volumes of deadwood, if it should be used to reach management goals towards natural tree succession.

### 4.5 Non‐uniformity in browsing susceptibility and intensity

Understory characteristics can have a significant influence of the future composition of forests, by moderating deer browsing patterns and shaping potential filtering effects among tree species (Begley‐Miller et al., 2014; Chollet et al., 2021). Silver fir is a highly relevant species for forestry (Schwarz & Bauhus, 2019), and our results show that young individuals of this species can be affected by associational susceptibility, associational resistance and conspecific neighbourhood effects simultaneously, demonstrating that variation in local understory vegetation conditions can have diverging effects on woody plants. High understory cover and the proximity and abundance of conspecifics reduced browsing intensity on *A. alba* through resource dilution and associational resistance. Comparatively, higher understory diversity led to associational susceptibility, presumably by increasing the overall attractivity of the resource patch for roe deer. Managing understory composition may aid to increase the resilience of managed forests against climate‐change‐related calamities.

### 4.6 Limitations and next steps

Like the literature, we did not find definitive effects of the studied variables on browsing intensity (Champagne, Dumont, et al., 2018; Kupferschmid et al., 2020; Milligan & Koricheva, 2013; Verheyden‐Tixier et al., 1998). This may be because these metrics alone are not yet sufficient to understand associational effects and their directionality. For example, in our study, plant species diversity increased the likelihood of individual fir trees being browsed. However, this might also depend on the overall plant diversity in the landscape and the opportunities roe deer have for resource patch selection, which in turn is affected by forest management (Hedwall et al., 2019; Márialigeti et al., 2016). In our study area, management for timber production excludes late successional forest stages which are typically rich in plant species (Scherzinger, 1996), likely leading to low overall understory diversity. We speculate that in a forest with higher large‐scale plant diversity, individual patches of diverse vegetation might not stand out as very attractive for roe deer, as many other similarly attractive sites are available, which would reduce the extent of associational susceptibility we found in our results. Consequently, larger scale patterns as well as local site conditions, for which forest management can be a strong driver, likely affect the extend and directionality of associational effects.

In our study area, high human disturbance in the forest, lack of natural predators, and intense hunting to control roe deer densities may have also influenced our result. Roe deer may trade‐offs between risk and resource selection in areas with high human disturbance (e.g. hunting, recreation; Bonnot et al., 2013; Borowski, Bartoń, et al., 2021; Gerhardt et al., 2013; Möst et al., 2015), and thus the relationship between roe deer abundance and browsing may be different in areas with lower human presence. Finally, associational effects also depend on the population density of the herbivore species (Vehviläinen & Koricheva, 2006). In our study area roe deer density is kept well below carrying capacity through culling. We speculate that in areas with higher roe deer density, forage might become limited, thus *P. abies* as well as *F. sylvatica* might be browsed more intensively. Similarly, where abundances of roe deer are lower than in this study, one might find associational effects on highly preferred woody plants as deer can be more selective.

Small‐scale neighbourhood relationships can affect herbivore resource use (Champagne et al., 2016; Holík and Janík., 2022), thus we might have failed to detect some effects of the understory as deer foraging decisions change depending on the distance to neighbouring plants. Moreover, we only assessed the browsing effect on woody plants clustered with conspecifics on a small spatial scale which might not show all occurring interactions between conspecifics.

Future studies should investigate further into the dependency of associational effects of understory vegetation on the underlying deer abundance as well as their preference towards different plant species. Specifically, it would be valuable to assess roe deer preferences among non‐woody species as well as the associational effects of plant guilds (herbs, grasses etc.). Here we are only assessing conspecific and total effects of the understory, but it is possible that specific groups of plant of high nutritional value are disproportionately driving roe deer forage selection. It would also be interesting to disentangle the impact of human presence and natural predation on roe deer browsing patterns, as these are other key drivers driving habitat use of roe deer. Furthermore, the influence of forage availability and quality on local browsing pressure should be assessed on multiple spatial scales to better understand the dependency of deer resource selection on local conditions, which would allow us to give more conclusive recommendations to managers.

## 5 CONCLUSION

In our study, we demonstrate that browsing on species that are of very low or high preference to roe deer is not affected by the characteristics of the understory. However, our study shows that understory characteristics are a strong driver for some woody‐plant species like for example *A. alba*. Specifically, our results show that woody plants can be exposed to association resistance, susceptibility and conspecific effects at the same time by different characteristics of the understory. Thus, forest managers should take understory characteristics into consideration when trying to counter browsing damage by roe deer. Increasing overall forage availability, possibly by opening the canopy, could for example contribute to reduce overall browsing damage and increase species richness in the canopy and consequently forest resilience long‐term. Unlike what one would expect, roe deer abundance was only related to browsing on some woody species like silver fir. Managers should be aware that browsing pressure is not solely dependent on deer abundance, thus culling alone, might not be an effective measure to reduce browsing pressure on all woody‐plant species. Instead, managers should aim to integrate management of food resources (e.g. by increasing overall understory cover) into their toolbox in order to decrease browsing pressure on young trees.

## AUTHOR CONTRIBUTIONS

**Sebastian Schwegmann:** Conceptualization (lead); formal analysis (lead); investigation (lead); writing – original draft (lead). **Martin Mörsdorf:** Conceptualization (supporting); formal analysis (supporting); investigation (supporting); writing – review and editing (supporting). **Manisha Bhardwaj:** Formal analysis (supporting); methodology (supporting); supervision (equal); writing – review and editing (equal). **Ilse Storch:** Conceptualization (supporting); funding acquisition (lead); supervision (equal); writing – review and editing (equal).

## FUNDING INFORMATION

This study was funded by the German Research Foundation (DFG), ConFoBi project no. GRK 2123.

## CONFLICT OF INTEREST STATEMENT

The authors declare no conflict of interests.

## Supporting information


Appendix S1

## Data Availability

Data is available in Dryad Digital Repository. https://doi.org/10.5061/dryad.7wm37pvzt.
