# Supplementary material for: Effects of understory characteristics on browsing patterns of roe deer in central European mountain forests
Source: Ecol Evol. 2023 Aug 14;13(8):e10431. doi: 10.1002/ece3.10431 (PMC10425343; doi:10.1002/ece3.10431)
Supplement: Supplementary file 1 — Appendix S1 [file ECE3-13-e10431-s001.docx]

# Appendix S1: Camera trapping yield for predictor of relative roe deer abundance

| Period | Positions | Roe deer events | Trapnights | Events per trapnight |
| --- | --- | --- | --- | --- |
| Spring 2019 | 126 | 1,973 | 6,580 | 0.300 |
| Autumn 2019 | 130 | 2,197 | 9,401 | 0.234 |
| Spring 2020 | 141 | 1,809 | 10,658 | 0.170 |
| Autumn 2020 | 141 | 2,563 | 12,416 | 0.206 |
| Spring 2021 | 141 | 2,212 | 11,262 | 0.196 |

# Appendix S2: Included candidate models (<dAIC2) for species specific generalized linear mixed models

Abbreviations: Ht: Height, DW: DW, Gr: Grouping, Co: Conspecifics, Y: Year. Values for cathegorical variables Grouping and Year in candidate models are not reported.

| ***Abies alba*** | **AICc** | **ΔAICc** | **weight** |
| --- | --- | --- | --- |
| -2.90Intercept+0.26RDeer+ 2.34Ht-1.99Ht²+0.44TotalWP-0.35UnderC+0.41UnderDiv+Gr-033Co+Y | 1103.31 | 0 | 0.171 |
| -2.90Intercept+0.27RDeer+ 2.35Ht-2.00Ht²+0.45TotalWP-0.35UnderC+0.39UnderDiv+Gr-0.34Co+0.10DW+Y | 1104.52 | 1.21 | 0.093 |
| -2.91Intercept+0.26RDeer+ 2.33Ht-1.98Ht²+0.43TotalWP-0.34UnderC+0.43UnderDiv-0.07UnderC*UnderDiv+Gr-0.30Co+Y | 1104.98 | 1.67 | 0.074 |
| -2.95Intercept+0.25RDeer+ 2.30Ht-1.93Ht²+0.32TotalWP-0.36UnderC+0.39UnderDiv+Gr+Y | 1105.22 | 1.91 | 0.066 |
| -2.91Intercept+0.27RDeer+ 2.34Ht-1.99Ht²+0.44TotalWP-0.34UnderC+0.40UnderDiv-0.07UnderC*UnderDiv+Gr-0.32Co+0.10DW+Y | 1106.20 | 2.89 | 0.040 |
| -2.55Intercept+Y | 1177.10 | 73.79 | 0 |
|  |  |  |  |
|  |  |  |  |
| ***Acer pseudoplatanus*** | **AICc** | **ΔAICc** | **weight** |
| -1.04Intercept-0.45RDeer+Y | 447.89 | 0 | 0.038 |
| -1.00Intercept-0.43RDeer+0.84Ht-0.76Ht²+Y | 448.53 | 0.64 | 0.027 |
| -1.02Intercept-0.44RDeer-0.11Co+Y | 449.14 | 1.25 | 0.020 |
| -1.02Intercept-0.44RDeer+0.09Ht+Y | 449.29 | 1.40 | 0.019 |
| -1.06Intercept-0.50RDeer-0.09DW+Y | 449.34 | 1.45 | 0.017 |
| -1.03Intercept-0.51RDeer0.90Ht-0.79Ht²-0.14DW+Y | 449.43 | 1.53 | 0.016 |
| -1.03Intercept-0.46RDeer-0.07UnderDiv+Y | 449.63 | 1.74 | 0.015 |
| -1.37Intercept-0.45RDeer+Gr+Y | 449.68 | 1.78 | 0.015 |
| -0.98Intercept-0.43RDeer0.90Ht-0.74Ht²-0.11Co+Y | 449.71 | 1.82 | 0.014 |
| -1.04Intercept-0.44RDeer-0.05TotalWP+Y | 449.74 | 1.85 | 0.014 |
| -1.04Intercept-0.45RDeer+0.04Ht²+Y | 449.82 | 1.93 | 0.013 |
| -1.03Intercept-0.45RDeer+0.03UnderC+Y | 449.89 | 1.99 | 0.011 |
| -0.99Intercept-0.45RDeer+0.85Ht-0.78Ht²-0.07UnderDiv+Y | 450.30 | 2.41 | 0.011 |
| -1.02Intercept+Y | 456.20 | 8.31 | 0 |
| 2.34 |  |  |  |
|  |  |  |  |
| ***Fagus sylvatica*** | **AICc** | **ΔAICc** | **weight** |
| -2.67Intercept-0.45Co+Y | 496.16 | 0 | 0.032 |
| -2.69Intercept-0.18Ht²-0.49Co+Y | 496.28 | 0.12 | 0.030 |
| -2.68Intercept-0.14Ht-0.48Co+Y | 496.90 | 0.74 | 0.022 |
| -2.68Intercept+0.12RDeer-0.19Ht²-0.45Co+Y | 497.16 | 1.00 | 0.020 |
| -2.67Intercept+0.11RDeer-0.42Co+Y | 497.19 | 1.03 | 0.019 |
| -2.67Intercept-0.21Ht²+0.12UnderC-0.52Co+Y | 497.42 | 1.03 | 0.017 |
| -2.68Intercept+0.50Ht-0.69Ht²-0.49Co+Y | 497.50 | 1.34 | 0.017 |
| -2.66Intercept-0.19Ht²-0.10UnderDiv-0.53Co+Y | 497.71 | 1.54 | 0.015 |
| -2.68Intercept+0.12RDeer-0.15Ht-0.45Co+Y | 497.78 | 1.62 | 0.014 |
| -2.66Intercept+0.08UnderC-0.47Co+Y | 497.81 | 1.65 | 0.014 |
| -2.66Intercept-0.08UnderDiv-0.48Co+Y | 497.82 | 1.66 | 0.013 |
| -2.78Intercept+Gr-0.42Co+Y | 497.98 | 1.82 | 0.012 |
| -2.67Intercept-0.18Ht+0.12UnderC-0.51Co+Y | 498.07 | 1.90 | 0.012 |
| -2.69Intercept-0.45Co+0.04DW+Y | 498.07 | 1.90 | 0.012 |
| -2.69Intercept-0.19Ht²-0.48Co+0.05DW+Y | 498.14 | 1.98 | 0.011 |
| -2.71Intercept-0.18Ht²+Gr-0.48Co+Y | 498.30 | 2.13 | 0.011 |
| -2.58Intercept+Y | 497.20 | 1.04 | 0 |
|  |  |  |  |
|  |  |  |  |
| ***Fraxinus excelsior*** | **AICc** | **ΔAICc** | **weight** |
| -1.40Intercept+0.46Ht-0.83UnderC-0.54UnderDiv+Y | 224.44 | 0 | 0.028 |
| -1.50Intercept+1.63Ht-1.05Ht²-0.92UnderC-0.45UnderDiv+0.40Co+Y | 224.57 | 0.13 | 0.026 |
| -1.48Intercept+0.54Ht-0.86UnderC-0.47UnderDiv+0.28Co+Y | 224.69 | 0.25 | 0.024 |
| -1.65Intercept+1.68Ht-1.11Ht²-0.77UnderC+0.48Co+Y | 224.78 | 0.34 | 0.023 |
| -1.62Intercept+0.51Ht-0.69UnderC+0.35Co+Y | 225.24 | 0.81 | 0.018 |
| -1.51Intercept+1.75Ht-1.34Ht²-0.74UnderC+0.44Co-0.29DW+Y | 225.41 | 0.98 | 0.017 |
| -1.53Intercept+0.52Ht-0.97UnderC-0.61UnderDiv+Y | 225.55 | 1.11 | 0.016 |
| -1.42Intercept-0.17RDeer+0.46Ht-0.74UnderC-0.48UnderDiv+Y | 225.64 | 1.20 | 0.015 |
| -1.39Intercept+1.07Ht-0.61Ht²-0.84UnderC-0.54UnderDiv+Y | 225.65 | 1.21 | 0.015 |
| -1.51Intercept+0.57Ht-0.67UnderC+0.32Co-0.28DW+Y | 225.90 | 1.46 | 0.013 |
| -1.55Intercept+0.55Ht-0.22TotalWP-0.89UnderC-0.46UnderDiv+0.37Co+Y | 226.02 | 1.58 | 0.012 |
| -1.56Intercept+0.40Ht-0.59UnderC+Y | 226.05 | 1.61 | 0.012 |
| -1.73Intercept+1.67Ht-1.09Ht²-0.22TotalWP-0.82UnderC+0.57Co+Y | 226.05 | 1.61 | 0.012 |
| -1.57Intercept+1.59Ht-1.00Ht²-0.19TotalWP-0.94UnderC-0.43UnderDiv+0.47Co+Y | 226.11 | 1.67 | 0.012 |
| -1.56Intercept-0.26RDeer+0.41Ht-0.54UnderC+Y | 226.16 | 1.73 | 0.012 |
| -1.40Intercept+0.38Ht²-0.77UnderC-0.52UnderDiv+Y | 226.19 | 1.75 | 0.011 |
| -1.64Intercept-0.17RDeer+1.67Ht-1.12Ht²-0.71UnderC+0.40Co+Y | 226.28 | 1.85 | 0.011 |
| -1.59Intercept+1.69Ht-1.07Ht²-0.26TotalWP-0.79UnderC+0.53Co-0.33DW+Y | 226.34 | 1.90 | 0.011 |
| -1.41Interceptt-0.21RDeer+1.21Ht-0.76Ht²-0.74UnderC-0.47UnderDiv+Y | 226.43 | 1.99 | 0.010 |
| -1.39Interceptt+0.48Ht-0.81UnderC-0.48UnderDiv-0.09DW+Y | 226.43 | 1.99 | 0.010 |
| -1.70Intercept+0.52Ht-0.22TotalWP-0.73UnderC+0.44Co+Y | 226.43 | 1.99 | 0.010 |
| -1.41Intercept+0.48Ht-0.06TotalWP-0.83UnderC-0.54UnderDiv+Y | 226.47 | 2.04 | 0.010 |
| -1.40Intercept+Y | 231.20 | 6.76 | 0 |
|  |  |  |  |
|  |  |  |  |
| ***Picea abies*** | **AICc** | **ΔAICc** | **weight** |
| -7.95Intercep-1.70Co+Y | 118.43 | 0 | 0.029 |
| -7.67Intercept+Y | 119.22 | 0.79 | 0.019 |
| -7.94Intercept+0.31Ht-1.51Co+Y | 119.39 | 0.96 | 0.018 |
| -7.83Intercept+0.39UnderC-1.87Co+Y | 119.53 | 1.10 | 0.016 |
| -7.69Intercept+0.39Ht+Y | 119.60 | 1.17 | 0.016 |
| -7.85Intercept+0.29RDeer-1.67Co+Y | 119.86 | 1.43 | 0.014 |
| -7.81Intercept+Gr-1.89Co+Y | 119.90 | 1.47 | 0.013 |
| -7.93Intercept+0.20Ht²-1.57Co+Y | 119.96 | 1.53 | 0.013 |
| -7.92Intercept-1.59Co+0.18DW+Y | 119.97 | 1.54 | 0.013 |
| -8.05Intercept+1.77Ht-1.42Ht²-1.51Co+Y | 120.04 | 1.61 | 0.013 |
| -7.81Intercept+0.20UnderDiv-1.60Co+Y | 120.26 | 1.83 | 0.011 |
| -7.67Intercept1.88Ht-1.42Ht²+Y | 120.28 | 1.85 | 0.011 |
| -7.66Intercept+0.27DW+Y | 120.32 | 1.89 | 0.011 |
| -7.73Intercept+0.27Ht²+Y | 120.34 | 1.91 | 0.011 |
| -7.76Intercept+0.35UnderDiv+Y | 120.68 | 2.25 | 0.010 |
| -7.67Intercept+Y | 117.60 | -0.83 | 0 |
|  |  |  |  |
|  |  |  |  |
| ***Rubus spp.*** | **AICc** | **ΔAICc** | **weight** |
| -0.14Intercept+2.55Ht-1.59Ht²-0.22UnderC+Y | 1563.67 | 0 | 0.029 |
| -0.16Intercept+2.57Ht-1.60Ht²-0.19TotalWP-0.25Co+Y | 1563.94 | 0.27 | 0.025 |
| -0.19Intercept+2.55Ht-1.59Ht²-0.22Co+Y | 1564.29 | 0.62 | 0.021 |
| 0.00Intercept+2.54Ht-1.58Ht²-0.20TotalWP+Gr-0.31Co+Y | 1564.29 | 0.63 | 0.021 |
| 0.00Intercept+2.53Ht-1.60Ht²-0.24UnderC+Gr+Y | 1564.48 | 0.82 | 0.019 |
| -0.10Intercept+2.56Ht-1.57Ht²-0.14TotalWP-0.19UnderC+Y | 1564.49 | 0.82 | 0.019 |
| -0.04Intercept+2.52Ht-1.57Ht²+Gr-0.28Co+Y | 1564.58 | 0.91 | 0.018 |
| -0.11Intercept+2.53Ht-1.58Ht²+Y | 1564.62 | 0.96 | 0.018 |
| -0.07Intercept+2.54Ht-1.60Ht²-0.17TotalWP+Y | 1564.84 | 1.17 | 0.016 |
| -0.13Intercept+0.13RDeer+2.56Ht-1.59Ht²-0.20TotalWP-0.25Co+Y | 1564.97 | 1.30 | 0.015 |
| -0.12Intercept+0.11RDeer+2.55Ht-1.59Ht²-0.21UnderC+Y | 1564.98 | 1.32 | 0.015 |
| -0.13Intercept+2.55Ht-1.59Ht²-0.22UnderC+0.09DW+Y | 1565.22 | 1.55 | 0.013 |
| -0.17Intercept+2.56Ht-1.59Ht²-0.16UnderC-0.11Co+Y | 1565.34 | 1.68 | 0.013 |
| -0.02Intercept+0.12RDeer+2.53Ht-1.59Ht²-0.20TotalWP+Gr-0.31Co+Y | 1565.36 | 1.70 | 0.012 |
| -0.14Intercept+0.12RDeer+2.54Ht-1.58Ht²-0.22Co+Y | 1565.43 | 1.77 | 0.012 |
| -0.15Intercept+2.54Ht-1.58Ht²-0.13TotalWP-0.22UnderC+Gr+Y | 1565.49 | 1.82 | 0.012 |
| -0.15Intercept+2.55Ht-1.59Ht²-0.22UnderC+0.04UnderDiv+Y | 1565.60 | 1.94 | 0.011 |
| 0.02Intercept+2.56Ht-1.60Ht²-0.20TotalWP-0.25Co+0.07DW+Y | 1565.64 | 1.97 | 0.011 |
| -0.14Intercept+2.57Ht-1.61Ht²-0.18TotalWP-0.09UnderC-0.25Co+Y | 1565.65 | 1.99 | 0.011 |
| -0.15Intercept+2.53Ht-1.58Ht²-0.15UnderC+Gr-0.18Co+Y | 1565.70 | 2.04 | 0.011 |
| 0.05Intercept+Y | 1791.50 | 227.83 | 0 |
|  |  |  |  |
|  |  |  |  |
| ***Sorbus aucuparia*** | **AICc** | **ΔAICc** | **weight** |
| -1.75Intercept+1.93Ht-1.66Ht²-0.28Co-0.24DW+Y | 457.12 | 0 | 0.036 |
| -1.67Intercept+1.97Ht-1.71Ht²-0.22Co+Y | 457.45 | 0.32 | 0.031 |
| -1.63Intercept+1.93Ht-1.67Ht²+Y | 457.60 | 0.48 | 0.028 |
| -1.62Intercept+1.96Ht-1.69Ht²-0.22TotalWP+Y | 457.61 | 0.49 | 0.028 |
| -1.75Intercept+0.14RDeer+1.90Ht-1.63Ht²-0.30Co-0.24DW+Y | 458.27 | 1.15 | 0.020 |
| -1.69Intercept+1.89Ht-1.62Ht²-0.17DW+Y | 458.41 | 1.28 | 0.019 |
| -1.67Intercept+1.92Ht-1.65Ht²-0.22TotalWP-0.17DW+Y | 458.41 | 1.29 | 0.019 |
| -1.67Intercept+0.15RDeer+1.95Ht-1.69Ht²-0.24Co+Y | 458.56 | 1.44 | 0.018 |
| -1.72Intercept+1.94Ht-1.66Ht²-0.13TotalWP-0.23Co-0.22DW+Y | 458.61 | 1.49 | 0.017 |
| -1.64Intercept+1.98Ht-1.72Ht²-0.16TotalWP-0.16Co+Y | 458.64 | 1.52 | 0.017 |
| -1.74Intercept+1.96Ht-1.69Ht²+0.10UnderDiv-0.28Co-0.25DW+Y | 458.73 | 1.61 | 0.016 |
| -1.62Intercept+0.13RDeer+1.67Ht-1.67Ht²-0.23TotalWP+Y | 458.86 | 1.73 | 0.015 |
| -1.64Intercept+0.12RDeer+1.65Ht-1.65Ht²+Y | 459.04 | 1.91 | 0.014 |
| -1.66Intercept+1.75Ht1-1.75Ht²+0.09UnderDiv-0.22Co+Y | 459.11 | 1.98 | 0.013 |
| -1.88Intercept+1.66Ht-1.66Ht²+Gr-0.28Co-0.24DW+Y | 459.15 | 2.03 | 0.013 |
| -1.50Intercept+Y | 477.00 | 19.88 | 0 |
|  |  |  |  |
|  |  |  |  |
| ***Vaccinium myrtillus*** | **AICc** | **ΔAICc** | **weight** |
| -2.30Intercept+0.38RDeer+1.00Ht-0.76Ht²-0.40TotalWP+0.46UnderC-0.62Co-0.13DW-0.16DW*Ht+Y | 2053.52 | 0 | 0.108 |
| -2.29Intercept+0.40RDeer+0.97Ht-0.73Ht²-0.36TotalWP+0.40UnderC-0.55Co+Y | 2053.83 | 0.31 | 0.092 |
| -2.23Intercept+0.38RDeer+0.90Ht-0.67Ht²-0.41TotalWP+0.45UnderC+Gr-0.63Co-0.13DW-0.16DW*Ht+Y | 2054.15 | 0.63 | 0.079 |
| -2.22Intercept+0.40RDeer+0.87Ht-0.65Ht²-0.36TotalWP+0.39UnderC+Gr-0.55Co+Y | 2054.45 | 0.93 | 0.067 |
| -2.30Intercept+0.39RDeer+0.98Ht-0.72Ht²-0.32TotalWP+0.37UnderC+0.12UnderDiv-0.48Co+Y | 2054.76 | 1.24 | 0.058 |
| -2.30Intercept+0.37RDeer+1.01Ht-0.75Ht²-0.37TotalWP+0.43UnderC+0.10UnderDiv-0.57Co-0.13DW-0.16DW*Ht+Y | 2054.78 | 1.26 | 0.057 |
| -2.29Intercept+0.39RDeer+0.96Ht-0.71Ht²-0.38TotalWP+0.42UnderC-0.58Co-0.10DW+Y | 2054.92 | 1.40 | 0.053 |
| -2.24Intercept+0.39RDeer+0.88Ht-0.64Ht²-0.33TotalWP+0.36UnderC+0.12UnderDiv+Gr-0.50Co+Y | 2055.47 | 1.95 | 0.041 |
| -2.24Intercept+0.37RDeer+0.90Ht-0.67Ht²-0.38TotalWP+0.43UnderC+0.10UnderDiv+Gr-0.58Co-0.13DW-0.16DW*Ht+Y | 2055.49 | 1.96 | 0.040 |
| -2.22Intercept+0.39RDeer+0.86Ht-0.63Ht²-0.39TotalWP+0.41UnderC+Gr-0.52Co-0.10DW+Y | 2055.54 | 2.02 | 0.039 |
| -2.32Intercept+Y | 2090.70 | 37.18 | 0 |
